# Death as the extinction of the source of value: the constructivist theory of death as an irreversible loss of moral status

**DOI:** 10.1007/s11017-023-09656-w

**Published:** 2024-02-08

**Authors:** Piotr Grzegorz Nowak

**Affiliations:** Institute of Philosophy, Jagiellonian University, Grodzka 52, 31-044 Kraków, Poland

**Keywords:** Brain death, Definition of death, Moral status, Constructivism, Utilitarianism, Intuitionism

## Abstract

In 2017, Michael Nair-Collins formulated his Transitivity Argument which claimed that brain-dead patients are alive according to a concept that defines death in terms of the loss of moral status. This article challenges Nair-Collins’ view in three steps. First, I elaborate on the concept of moral status, claiming that to understand this notion appropriately, one must grasp the distinction between direct and indirect duties. Second, I argue that his understanding of moral status implicit in the Transitivity Argument is faulty since it is not based on a distinction between direct and indirect duties. Third, I show how this flaw in Nair-Collins’ argument is grounded in the more general problems between preference utilitarianism and desire fulfillment theory. Finally, I present the constructivist theory of moral status and the associated moral concept of death and explain how this concept challenges the Transitivity Argument. According to my view, brain death constitutes a valid criterion of death since brain death is incompatible with the preserved capacity to have affective attitudes and to value anything.

## Introduction

One of the less popular approaches within the definition of death debate defines death in terms of moral status [1–6]. In contrast to the proposal of defining death in purely biological terms (a mainstream position in the debate [7–11]) or by utilizing theories of personal identity [12, 13], ‘the moral view on death,’ as I will call such an approach, remains relatively undeveloped. This article aims to address this gap by answering the Transitivity Argument formulated against the moral view on death.

The motivation for the moral concept of death arises from the challenges posed to the definition of death by organismal pluralism in theoretical biology [6, 14–17]. While I briefly touch upon this difficulty in the next section below, the main goal of the paper is pursued in the following sections, where I elaborate on the moral view of death as “an irreversible loss of A’s moral status (whatever A is precisely)” [5, 6, p. 511]. Accordingly, I argue that the concept of moral status needs to be accounted for through the distinction of direct and indirect duties, and this distinction can be best explained by utilizing constructivism [18–21].

## Organismal and death pluralism

In this section, I will provide a brief summary of the argument presented in two of my recent articles [6, 30]. The main problem for the supporters of the mainstream view of death—as the extinction of an organism – stems from the fact that, contrary to what most lay people believe, a cat or human in a certain physiological state could be considered both alive and dead as an organism according to the most up to date theoretical biology [14–17]. In other words, when trying to determine whether an entity is alive or dead as an organism, one may receive conflicting answers that are all technically valid. For instance, the functional developmental concept defines an individual animal as one that progresses from ovum to ovum [22–24] and requires functional integration for an entity to be considered an individual organism [17]. The immunological concept considers an organism to be a collection of elements that do not elicit hostile immunological reactions between each other [25–27]. In contrast, the classic evolutionary concept defines an individual organism as capable of reproduction and participating in natural selection [28].

These different concepts present a challenge, as a single body including a brain-dead patient may be considered both alive and dead at the same time under various sub-theories of biology [29]. Subdisciplines of biology (e.g., immunology, developmental biology, evolutionary biology) offer equally valid but distinct perspectives on what constitutes an organism and its end. I will term this problem the ‘organismal pluralism problem.’

The organismal pluralism problem does not necessarily undermine the principle of non-contradiction or scientific realism. Instead, it challenges the formulation of the question of ‘Whether x (e.g., brain-dead patient) is an organism?’ by suggesting that it is ill-formulated. To determine an entity’s organismal status, one must first specify a relevant subdiscipline of biology. If one insists on a general answer to whether x is an organism, the response would be: ‘Yes and no. Yes, under such and such a branch of biology. No, under such and such branch of biology.’^1^

The “organismal pluralism problem” is often overlooked by supporters of the mainstream view in the definition of death debate, such as James Bernat [8, 10, 31], Alan Shewmon [9, 29, 32], members of the President’s Commission for the Study of Ethical Problems in Medicine and Biomedical and Behavioral Research [33], and members of the President’s Council on Bioethics [34]. These authors and many others [e.g., 7, 11, 35–43] have assumed throughout the history of the definition of death debate that a brain-dead patient under artificial support might be unanimously determined to be either alive or dead as an organism.

The plurality of deaths is problematic for traditional ethics and law since throughout history death has been considered to be a unified state. Yet, suppose a given body in a particular condition might be alive and dead as an organism. In that case, it is hard to imagine how, for example, a physician considering treatment options would proceed. Moreover, in ethics and law, the term “murder” essentially refers to wrongful killing [44]. If killing is defined as causing biological death, there is a dilemma: removing the heart from a brain-dead patient could be considered both killing (e.g., according to the immunological concept of an organism [6, 17, 25, 26]) and not killing (e.g., according to the functional developmental concept [17]). Both views have valid biological justifications but offer different perspectives. To uphold the idea that murder is objectively wrong, one needs a sense of ‘death’ that is different from the biological one and that is compatible with preserving notions like ‘murder’ to consistently describe and morally evaluate human behavior.

## Death as a thick moral concept

This article will not present new arguments to strengthen my previous claims on organismal pluralism [6, 30] but instead will focus on developing a sense of ‘death’ that preserves traditional moral concepts like ‘murder.’

The sense I refer to is the moral concept of death, which predates the modern concept of an organism [14, 45]. The idea is that the word ‘death,’ besides its purely biological ambiguous sense defined within up-to-date biological theories of an organism, also has a moral meaning. It is a thick moral concept, and in this regard, similar to words like ‘health’ and ‘disease.’ As James Lindemann Nelson says:

To be ill is not merely to have something going on in one’s body that is out of step with species design as laid down by evolution: it is to have something going on that is wrong, one that reduces our chances of being free of pain and unhindered our ability to pursue what we judge to be good. If our species design were to leprosy and die at fifty, leprosy would be an illness nonetheless. In this respect, health and illness language follow the pattern of thick moral concepts such as ‘brutality’ [46, p. 315].

The meaning of ‘death’ is similar to the thick moral meaning of ‘illness.’ To paraphrase Nelson, one probably does not understand the word “death” in everyday out-of-laboratory context if one “meets the news that another person is dead with perfect emotional indifference, and there is not some reason that explains this response—burnout, or bad character, or something of the sort.” [cf. 46, p. 313].

What is peculiar about thick moral concepts is that they refer to natural facts, which are also normative facts. These concepts undermine the Humean distinction between facts, which, according to this distinction, were non-normative, and values, which were normative but were not facts. What kind of fact is at stake when it comes to death? I believe that in a moral sense, ‘death’ means a state in which one cannot be harmed or benefited. Thus understood, death is a natural event since human vulnerabilities to harms and benefits are constituted by natural properties like sentience. Yet death, according to such an understanding, is also an event that has a normative sense; if someone becomes dead, one’s obligations that were grounded in the person’s capacity to have wellbeing come to an end.

In this sense of death, one can say that people are dead. One can also talk about inanimate material that it is dead and has always been dead in this way. In this perspective on death, the distinction of being an organism or not does not matter.

Robert Veatch proposed a definition of death in moral terms that aligns quite closely with my own [47 cf. 2, 3, cf. 48]. His idea was to “define the word death as the name applied to the category of beings who no longer have full moral standing as members of the human community with all the rights of that community (including the right not to be killed)” [3, p. 9]. Veatch emphasized that the moral sense of being dead alters the evaluation of behaviors, such as burial, grief, and organ retrieval. These behaviors are *prima facie* forbidden for the living but allowed for the dead [cf. 1, 2]. However, in contrast to Veatch, I believe that there is no need to associate a moral sense of death with the loss of ‘full moral standing’ in which only rights are grounded. Such a narrow perspective makes the ethical concept of death exclusive and nonapplicable to many animals. Rather, the word death could be understood as “an irreversible loss of A’s moral status (whatever A is precisely)” [5, p. 512]. Accordingly, in the following sections, I will take a closer look at the concept of moral status (sometimes, as in the case of Veatch, called moral standing) and the associated moral concept of death.

## Moral status

Moral standing, more frequently referred to as moral status, is defined by Agnieszka Jaworska and Julie Tannenbaum in the following manner: “An entity has moral status if and only if it or its interests morally matter to some degree for the entity’s own sake” [49, p. 242]. This understanding of moral status is a matter of widespread ethical consensus [20, 50–53]. What is crucial for my further investigation of death, however, is an adequate grasp of what is at stake when one contrasts things that matter morally *for the entity’s own sake* in the above definition with things that only matter *because of some consequences for other beings*.

I will explain the meaning of ‘being important for the entity’s own sake’ with reference to Mary Anne Warren's writings on moral status [51]. For simplicity, I will call all acts that are morally important for the entity’s own sake ‘direct obligations.’ Meanwhile, all the acts that are done to some entity and which do not matter to that entity’s own sake but matter to some other entity’s sake, I will term ‘indirect obligations’ [4, 5]. Warren writes:

An example may help to clarify this distinction. Suppose that you go on vacation, leaving your house in the care of a friend, who then sells your kitchen appliances and absconds. A moral wrong has evidently been committed; but it is obviously a wrong against you and not against your stove and refrigerator, which do not have moral status. Had you, on the other hand, left your pet pig in the care of a friend, who then sold it to a meat packing plant, then it would have been less clear that a wrong had been committed only against you. And if you had left your baby with a friend, who then sold him or her to a black-market baby broker, almost no one would doubt that a wrong had been committed not only against you but also against the child [51, p. 10].

Warren's examples help ground the distinction between direct and indirect duties intuitively. Further intuitive examples will aid in a deeper analysis of this distinction, but should not stop at the level of intuitions. So, one could say that I have a direct obligation not to kill my friend or inject them with hypnotic drugs without their consent. If I act contrary to this obligation, I do something that matters morally for “their own sake.” On the contrary, according to Mary Anne Warren, the entity that lacks moral status, such as kitchen appliances and absconds, might be treated as one pleases, or indirect obligations referring to these entities could be in place. For example, I might do something wrong to the owner of a stove if I destroy it.

## The transitivity argument

What has been said up until this point might be summarized with the following reasoning:

(P1) A’s death is as ‘an irreversible loss of A’s moral status (whatever A is precisely)’

(P2) A’s moral status is constituted by whatever it is that grounds all the direct obligations that are in place towards A.

(C) A’s death is an irreversible loss of whatever it is that grounds all the direct obligations that there are towards A.

To specify death further, I must explore the foundation of moral status. Let me start with Robert Veatch’s proposal, since he was most explicit in associating death with the loss of moral status. According to his model, roughly speaking, the integration of consciousness with a physical body constitutes the basis of moral status, and the irreversible loss of such integration equals the irreversible loss of moral status and *a fortiori* equals death [3, 54]. Yet, the problem is that Veatch simply states that the irreversible loss of the integration of consciousness with a body constitutes a loss of moral status without fully justifying this stance [55].

Now consider the plausible justification of such a view through hedonism, which asserts that all the things that impact conscious states like pleasure and suffering matter. According to hedonism, all sentient creatures possess moral standing, while all non-sentient beings lack it. Thus, direct obligations can be grounded only in sentience. The view linking moral status and the criterion of death to consciousness (understood as the capacity to feel pleasure and pain) seems straightforward. However, Michael Nair-Collins' ‘Transitivity Argument’ challenged it [55].

The main thrust of his argument is this: if the standard way of behaving toward end-stage dementia patients, that is, honoring their advance directives regarding their treatment (the so-called extension view [56]), is not morally misled, then hedonism fails to capture all of the possible grounds for moral status. And if hedonism fails to capture all of the grounds for moral status, it might be that brain-dead patients, although irreversibly unconscious, possess moral standing and are therefore alive. I will now more closely examine the details of the Nair-Collins’ Transitivity Argument. Consider a standard case of the advance directive:

When she was competent, Desiree explained to her family and physician that she would find it a grave affront to her sense of personal dignity to be kept alive in a condition when she could no longer recognize her family and friends. She explained that once she reaches this stage of cognitive decline, she wanted to be kept comfortable by being fed by mouth and having pain medications if indicated, but she refused any other life-sustaining treatments, including antibiotics or artificial nutrition [55, p. 547].

Suppose Desiree has lapsed into a state that fulfills the description written in her living will. As Nair-Collins explains, honoring the choice of Desiree can hardly be justified on the grounds of hedonism. Given her level of cognitive decline, she is plausibly unable to experience subtle psychical satisfaction from the fact that her life could end according to her sense of personal dignity. Instead, she can only now experience simple physical pleasures and pains. For the sake of argument, Nair-Collins assumes that Desiree’s life, if continued, would be full of simple physical pleasures (such as those caused by a backrub). If this is the case, there are no hedonic reasons supporting the decision to withhold the treatment of pneumonia with antibiotics [cf. 57]. Thus, if dishonoring Desiree would harm her, it must be for reasons other than her hedonic status.

Such a reason can be indicated by appealing to preference utilitarianism and the desire-fulfillment theory (which is, in fact, one of the elements of preference utilitarianism). Desire fulfilment theory, as Derek Parfit defines it, claims that “what would be best for someone is what would best fulfil his desires throughout his life” [58, p. 492]; while preference utilitarianism, in turn, upholds that each agent has a duty to maximize desire satisfaction in the world [59]. As Nair-Collins argues, to show why disrespecting Desiree’s will harms her, one might refer to investment interests, which are only based on the preferences or desires that the person is invested in (in the following, I will use the terms ‘desires’ and ‘preferences’ interchangeably) [55, 56, 60]. Although Desiree, according to Nair-Collins, can no longer formulate any new desires, she had preferences in the past and was invested in their achievement. Many philosophers, including, among others, Feinberg [61], Dworkin [62], and Regan [60], believe that such past desires are still morally binding. They are said to be binding even though people in a similar situation to Desiree can no longer confirm their previous will or formulate a new one. Accordingly, they are not aware of the fulfillment or deprivation of their desires.

I now move to the critical part of the Transitivity Argument, which aims to prove that brain-dead patients are considered alive if death is defined as an irreversible loss of moral status. This reasoning relies on comparing obligations toward three patients:

Daniel is in a state of end-stage dementia. He is no longer communicative and does not respond in purposeful ways to speech or to the presence of his caregivers; however, he does clearly react to noxious stimuli and shows signs of pain […]. Veronica is in a reliably diagnosed permanent vegetative state. Thus, for the purpose of this exercise, assume that Veronica is entirely unaware of self and surroundings; Veronica is biologically alive, but lacks sentience. [...] Finally, Christine is in a state of irreversible apneic coma satisfying diagnostic standards for brain death. […]. Christine, like Veronica, is biologically alive but irreversibly unconscious [55, p. 534].

In line with Nair-Collins' reasoning, consider Daniel in a situation similar to Desiree’s as described above. He possesses an advance directive stating that life-sustaining treatment should cease if he no longer recognizes his family, which is now the case. Furthermore, like Desiree, Daniel's current life involves simple sensual pleasures without significant pain.

Nair-Collins highlights that under the standard view in bioethics, one would be obliged to withhold antibiotics for Daniel’s pneumonia, despite knowing that his continued life would be filled with simple physical pleasures. The question arises: how can one have this obligation when Daniel's hedonic capacities do not seem to justify it? Nair-Collins asserts that the obligation towards Daniel can be based on Daniel’s past desires (which constitute investment interests) and autonomy. However, if Daniel's past preferences can justify this obligation, why cannot similar preferences serve the same purpose for Veronica and Christine? If obligations exist for Daniel which are not based on his sentience, why deny such obligations for Veronica and Christine? All three lack the ability to form new desires according to Nair-Collins. If obligations toward Daniel are indeed rooted in his past desires that constitute investment interests, then the same principle should equally apply to Veronica and Christine. If there are such obligations, then Veronica—and, most importantly, Christine—still have moral status and are morally alive.

To solve the problem posited by Nair-Collins, I need to analyze why the fulfillment of desires in cases like that of Daniel, Veronica, and Christine is normative and how it is so. The rebuttal of Nair-Collins’ argument has a lot to do with recognizing that the justification for the normativity of desires proposed by preference utilitarianism is unreliable. Essentially, there is a fundamental normative difference between frustrating the desire of Daniel on the one hand and dishonoring the will of Veronica or Christine on the other. Only in the first instance does one infringe on direct duties, while in the second, one infringes on indirect duties. I will show why this is so in the following sections.

## Intuitionism and constructivism

So, why is the satisfaction of desire normative, and in what way is it so? Usually, ethicists including preference utilitarians say that one intuitively sees that fulfillment of desires is good when one is confronted with some thought experiments like the famous Nozick’s experience machine [63]. Unfortunately, this intuitionist approach usually does not analyze the relation between the author of a preference and the preference itself [21, 64, 65]. That is, it does not say nor emphasize that fulfillment of desires is good *because someone has created the desire*. Accordingly, such intuitionist justification creates an impression that there could be free-floating desires generated out of nothing and by no one.

Is there an alternative to the intuitionist approach? I believe so. By relying on scientific knowledge, one can categorize entities in the world into two kinds based on their natural capacities. The members of the first kind manifest evaluative attitudes, while the members of the second kind are incapable of any valuation [19, 20, 66, 67]. What does it mean that the entities of the first kind manifest evaluative attitudes? It means nothing more than that they perform some affective attitudes [21]. They are capable of feeling the world as pleasurable or painful; they are capable of creating desires; of reflecting on their pains, pleasures, and desires; and of endorsing some of their pleasures, pains, and desires while revolting against some of their different pains, pleasures, and desires. In what follows, I will call the third kind of valuation ‘practical reflection’ and entities capable of performing some evaluative attitudes ‘valuers.’ The entities of the second kind do not perform affective attitudes and can only be objects of valuation. I will call the entities of the second kind ‘pure objects’ [cf. 68].

What more can be said about valuation? The important thing to notice is that all types of valuation are in fact a kind affection. ‘Simple valuers’ (e.g., lizards) only feel pain and pleasure which is pure affection and nothing more. On the other end of the valuing spectrum are persons as ‘complex valuers.’ They not only value things based on pleasurable and painful experiences but also form preferences, endorse some of them, and revolt against others. One can conceive ‘desiring’ as anticipating with some affect some activity to be done or event to take place [69, cf. 70–75]. The most advanced type of valuation is practical reflection, which is peculiar to persons. As Harry Frankfurt writes, “Besides wanting and choosing and being moved to do this or that, men may also want to have (or not to have) certain desires and motives. They are capable of wanting to be different, in their preferences and purposes, from what they are” [76, p. 128]. By endorsing certain desires, one acknowledges and accepts them. One allows these desires to direct one’s actions and have some positive affect associated with the thought that this desire will produce one’s actions [76]. Analogously, one can accept through practical reflection some of one’s pleasures and pains.

## The normativity of desires

Given the constructivist ontology of morals proposed herein, I will now look more closely at the normativity of desires and preferences. When backed by intuitionist moral epistemology, preference utilitarianism does not offer any explanation or justification for the normativity of preferences besides the alleged intuitively perceived *sui generis* goodness of their fulfillment. Preference utilitarianism also provides no theoretical apparatus to divide direct and indirect duties.

In the constructivist approach however, desire fulfillment's significance is explained by the existence of subjects as authors of valuation. All valuers have evolved to display affective attitudes towards the world, themselves, and others [21]. Valuers and their affective attitudes are essential for the existence of values in the world. Therefore, valuers, as creators of all values, hold remarkable worth. They condition the existence of the whole of morality. Following Christine Korsgaard, this role can be identified with so-called ‘unconditional value’ [66] since this role is about conditioning the existence of all normativity in the world.

Given this constructivist analysis, one can also say that the value of the fulfillment of desire is extrinsic—it is sourced in the valuer who conditions it [18, 68]. In other words, fulfilling desires is good because someone created them. It is not good *simpliciter*. Preferences require an author, and the constructivist moral theory properly acknowledges the author’s role. As will be shown, the constructivist explanation of the normativity of desires plays a crucial role in handling Nair-Collins’ Transitivity Argument.

## Valuation as constructing the borders of the self

I will now return briefly to the distinction between direct and indirect duties. But before I do so, I need to emphasize that the constructivist view on normativity comes with a theory of the identity of valuers. As Korsgaard says, “all value is tethered…” [20, p. 33]. There are no free-floating attitude-independent values as “[importance] is tethered to the creature to whom the thing in question is important, and it cannot be cut loose from that creature without ceasing to be important at all” [20, p. 32]. Values are ‘born’ to the world through valuers.

But values also constitute the borders of valuers. Consider the case of the lizard, which is burned during a meadow fire. Sentience is a capacity to actively demarcate the borders of oneself in such case. A lizard differentiates between its pains and pleasures and events that are neither pleasurable nor painful. A particular lizard constitutes its borders by feeling some affective sensation when it is, for example, burned or fed. It feels and therefore triggers the difference of whether it is burned or fed or if some other lizard is burned or fed. An affective sensation of being burned and fed is a mechanism constituting the borders of a concrete simple valuer and things that happen outside its borders. The particular lizard’s end lies there when it cannot be hurt nor pleased anymore.

Constructivism, as opposed to the classic utilitarian sentience-based criterion of moral status [77–80], allows one to recognize that pain and pleasure serve as a border-constituting mechanisms. Pain and pleasure not only serve as such mechanisms in the case of lizards but also in those of human beings. As Marya Schechtman says, “we all know the difference between fearing for our pain and fearing for the pain of someone else. The difference here consists not in degree—I may care more about the pain of my beloved than about my own—but in kind” [81, p. 52]. Utilizing Jeff McMahan’s terminology, one can say that there is something like egoistic concern that informs the particular valuer’s borders [12]. This kind of concern informs the distinction between a given valuer and all the stuff that is external to them, not only in case of simple valuations like feeling the word pleasurable or painful but also in case of more complex valuations like having desires and practical reflection. In a word, one knows the difference between caring for the fulfillment of one’s desires and the fulfillment of others' desires.

Now, consider this phenomenon from the neuro-cognitive or neuro-biological perspective. Neurosciences still have unanswered questions about consciousness, with competing theories like biological, higher order-thought, and global workspace/information integration theories [82]. Yet, important empirical questions remain unanswered. For example, as Melanie Boly et al. point out in their review article on the advances in neurosciences, one question is “whether specific cell types (e.g., deep vs. superficial pyramidal cells, Von Economo neurons, etc.) are more important for consciousness than others? Do consciousness-related neurons have specific morphologies and/or molecular properties, or is their connectivity the critical factor?” [82, p. 6].

However, neuroscience indicates with a high level of certainty that first-person awareness in adult humans—including pain, pleasure, and emotions—is impossible without functioning cerebral hemispheres. Given the review of scientific work, what is essential for awareness is “fronto-parietal connectivity and (…) ‘top-down’ processes, both of which enable information to travel across distant cortical areas effectively” [82, p. 1].

Given these findings, one can cautiously conclude that valuing as a process based on first-person affective attitudes is an activity in which cerebral hemispheres play an essential role. First-person affective experience in adult humans is probably impossible without cerebral hemispheres [83–86]. The endocrine system also plays an integral part in one’s valuations since it produces and releases hormones like testosterone, adrenaline, progesterone, and many others, significantly impacting the subjective quality of one’s affective experience [87]. Yet, absence of cerebral hemispheres means one cannot experience the impact of testosterone on mood. There are rare exceptions, though, like in newborns where neuroplasticity allows awareness despite lacking functioning cerebral hemispheres. In such cases, consciousness relies on a functioning brain stem. [12, 88, 89, cf. 90].

## Unconditional value, direct vs. indirect duties, and death

Now I must revisit the direct and indirect duties distinction. All valuers play a unique role as sources of normativity. Therefore, one can qualitatively distinguish acts that affect the status of valuers as such and acts that are done to facilitate or impede valued states of affairs [cf. 20]. Acts of the first type fulfill or infringe on direct duties, while actions of the second type meet or infringe on indirect duties. Utilizing the Kantian-Korsgaardian terminology, one can say that acts that affect the status of valuers target unconditional value. They achieve this through various means, like preserving, modifying, or destroying the ability to value. An example of infringing direct duty is injecting someone with a hypnotic drug without their consent. In this way, one suspends one’s ability to value by feeling, creating desires, or engaging in practical reflection. When it comes to the infringement of an indirect duty, a good example might be when someone burns another against their will. In such a situation, the affected person feels pain and is therefore valuing the fire negatively; moreover, their desire not to be burned is unfulfilled; and, the person perceives their pain and deprivation of their desire not to be burned as bad. Yet, being burned does not impact the person’s mere capacity to value.

Note that non-constructivist theories lack the ability to distinguish acts that infringe direct duties as a separate category. For them, injecting someone with hypnotic drugs might be deemed wrong based on depriving pleasure, causing suffering or pain, or opposing one's preferences. However, the moral significance of suspending one's evaluation capacities is irrelevant in non-constructivist theories.

Consider now how the constructivist perspective clarifies the distinction between direct and indirect duties for Daniel, Veronica, and Christine. With this analysis in place, one can challenge the Transitivity Argument and defend the view that:

1. Brain death is death.
2. End-stage dementia is not death.
3. A persistent vegetative state, if it could be determined beyond any doubt (which is not the case), is death.

This position is justified if one can demonstrate that Daniel persists as a valuer while Christine and Veronica no longer do. If one’s actions can potentially affect Daniel's valuating capacities, direct obligations exist to him, along with his moral status and life in the moral sense.

It is obvious that Daniel remains a valuer in end-stage dementia and one’s actions can impact his valuing nature, leading to the compliance or breach of direct obligations towards him. Daniel’s sentience reveals his nature as a valuer. He still demarcates his borders through his ability to feel pain and pleasure. He creates values through his sentience. He still feels the difference between his pains and pleasures and things outside his borders. Importantly, this affective consciousness of his boundaries is, in essence, the same kind of psychological faculty that he manifested when he could understand and feel the difference between the fulfillment of his more complex life plans and the fulfillment of life plans of others. That is because all valuation has the core element of conscious affection, either simple as in the case of Daniel and lizards, or complex as the affection present in desiring or practical reflection. Considering life's evolutionary process, it is conceivable that there are no sharp boundaries between simple and complex valuations; the contrast is more of a gradual continuum [21]. However, what does make a difference is the presence or absence of any capacity to value in the given subject.

Given the constructivist view of valuation as a process of demarcating one’s borders, Daniel persists as the same individual valuer despite a significant reduction in the spectrum of available valuations due to dementia. The preferences regarding Daniel’s present fate, formed in the past, come from the same valuer with the same egoistic center, despite his current valuations being less complex than before.

Now, compare Daniel’s situation with that of Veronica and Christine. Like Daniel, Veronica and Christine cannot formulate and endorse desires but they have lost the ability to evaluate states through pleasure or pain, and no affective characteristic applies to them. One cannot affect their way of valuing things since they can no longer (and will never be able to) perform this vital activity. In contrast, one can still influence Daniel’s capacity to value. For that reason, one can legitimately say that there are no more direct obligations towards Veronica and Christine; thus, they have lost their moral status and are morally dead whereas Daniel is morally alive. It does not mean that one can never act wrongly if one does not honor Veronica and Christine's wishes regarding the treatment of their bodies. However, such wrongdoings do not violate any direct duty towards their bodies, as they are no longer valuers. Such misconduct may be considered posthumous harm [91], which is a harm done to the person from the past (a controversial concept), or harm to society [92], including, primarily, harm to the relatives of the person who is now morally deceased.

When Christine and Veronica’s situation is contrasted with Daniel’s, one sees that respecting Daniel’s wish to cease life-sustaining treatment is based on his affective nature as a valuer, still present despite significant compromise. His affection now exists solely as sentience, yet even in this form, it signifies the preservation of Daniel as a single valuing subject enduring over time. Moreover, withdrawing life-sustaining treatment targets his core ability to value. This conduct is performed to stop his simple valuations (which are based on pure sentience). It is an act of putting an end to his existence in the form that Daniel evaluated as undesirable. By honoring his wish, one fulfills his past desire to cease being a valuer when his way of valuing the world becomes devastatingly modified to an extent that was unacceptable to him [cf. 93, 94]. One is modifying his valuating capacity in a way that was authorized by himself as a valuer and that means that one is fulfilling their direct duty toward Daniel.

To sum up: Veronica and Christine have irreversibly lost their nature as valuers, losing their moral statuses and becoming morally dead. In contrast, Daniel has not lost his nature as a valuer and thus his moral status remains. He is still the same valuer as he was as a competent person, albeit damaged, and therefore he is morally alive. However, the diagnosis of a persistent vegetative state (the case of Veronica) and irreversible unconsciousness under conditions other than brain death currently remains uncertain in real-life scenarios [83, 90, 95].^2^

In contrast, one can be sufficiently sure that brain-dead patients, with no blood flow to the brain, cannot experience the world, formulate desires, or reflect, making them morally dead.

Of course, there are rare reports of patients who have regained conscious awareness after the diagnosis of brain death, such as the famous case of Zack Dunlap [96]. In all these cases, there is insufficient evidence to confirm that the death determination was formally appropriate, except for one exception to be discussed later [97]. The situation is therefore distinct from diagnosing the vegetative state, where clinical tests, even when properly conducted by skilled neurologists, may yield a positive diagnosis despite a frequent presence of neurological correlates of conscious awareness visible through neuroimaging technology. As mentioned above, there might be one exception when it comes to brain death, the one event when the patient possibly regained some level of conscious awareness after the formally appropriate determination of death by currently accepted neurological criteria. This is the case of Jahi McMath.

She was diagnosed as brain-dead in 2013, but a few years later, her family presented videos of Jahi responding to simple commands like ‘give us a thumbs up.’ Moreover, Alan Shewmon has personally confirmed the family observations: “6 days before she died, I visited her in her hospital room and observed a (non-myoclonic) right arm movement in response to her mother’s command to move that arm. (There had been no spontaneous movements of any kind up that point or for the rest of my visit, so it was clearly not a chance coincidence of a random baseline movement.)” [98, p. 169, cf. 99–101]. Alan Shewmon argues that Jahi was never brain-dead but instead suffered from global ischemic penumbra mimicking brain death. According to him her brain showed partial regeneration to the minimally conscious state due to minimal undetectable blood flow. Note however, this interpretation of the case is disputed [102, 103].

Whether Jahi regained consciousness or not, the situation with brain death is distinct from Adrian Owen's findings on patients diagnosed with a vegetative state. The certainty of the loss of conscious awareness, including sentience, in patients diagnosed with brain death is significantly higher than that in a diagnosis of a vegetative state. The certainty in this context is also higher than in the case of anencephalic infants, who often possess functioning brain stems. These could, in theory, form the basis for conscious experiences due to neuroplasticity in newborns. But of course even in the case of brain death, one has only a lack of evidence for conscious awareness rather than positive evidence of unawareness. Moreover, the lack of evidence for the presence of some phenomenon cannot be considered as entirely certain evidence of the nonexistence of this phenomenon. Yet, one cannot assume everything is capable of affection because one lacks positive proof of the absence of awareness. Practical reasons demand reasonable criteria.

Given the upshot of the examples from neuroscience, I believe the risk of false diagnosis of loss of capacity for conscious awareness in the case of brain death is so small that one should accept it. It is especially the case given that the circulatory criterion of death might be less reliable here, as some investigations on the brain tolerance for warm ischemia show [104, 105, cf. 106]. This does not mean no improvements can be made in diagnosing brain death and efforts to enhance such diagnosis should be pursued but practically improving tests for brain death is beyond the scope of this research. However, I concur with Christos Lazaridis and Fernando D. Goldenberg, who concluded from an analysis of many cases of false positive diagnoses of brain death that there is “the need to demonstrate absence of brain circulation to make a determination of death by neurologic criteria” [107, p. 210]. Intracranial blood flow tests should be obligatory, and not anymore treated as ancillary according to these neurologists. Such a solution will likely substantially decrease the risk of a false positive diagnosis of brain death, though not entirely (for the discussion of limitations of blood flow studies, see: [108]).

Finally, I would like to clarify how the constructivist theory of death presented here compares with the constructivist view on death advocated by Robert Truog, Franklin Miller, and Shema K. Shah [109]. Contrary to my position, these authors believe in a singular real biological death. It is a moment “…when the entropy-increasing forces have irreversibly exceeded those that are resisting this process” [109, p. 70]. According to them, in artificially sustained brain-dead patients, the critical moment of entropy increase surpassing the resisting forces has not yet arrived. Thus, these authors conclude that brain-dead patients are, in fact, alive. Despite acknowledging the falsehood of brain death as a criterion of death, they recommend its use due to its utility in facilitating organ donation. In other words, according to Miller, Truog, and Shah, it is a fiction that brain death is death [cf. 110], albeit a useful legal fiction. Now since a legal fiction can, in a sense, be understood as a social construct [34], Miller, Truog, and Shah’s perspective can be termed ‘a constructivist view on death’ [48].

Their version of constructivism differs significantly from mine. Miller, Truog, and Shah’s approach allows for broad flexibility in selecting a criterion of death, suggesting that any fictional standard can be chosen if it serves a useful purpose for society. Besides their position there are also other constructivist approaches conceiving social death as a matter of loosely restrained choice [48, 111].

In contrast, the constructivist view proposed herein starts from the assumption that death not only has a biological meaning and that this word has a different meaning in an everyday context. In other words, it is a morally thick term and death means the irreversible loss of a given entity’s moral status. There is a nonarbitrary truth to be discovered as to what grounds moral status. The truth of the matter is the following: there can be no moral status without evaluative capacities, and evaluative capacities are enough to ground moral status. Death is not a matter of arbitrary choice.

## The causal relation between death and moral status

In the following sections I consider three objections to the view I have presented. I will start with the following objection: “the loss of moral status is a *consequence* of death understood as cessation of an organism. Stated differently, cessation of an organism is the *cause* of the loss of moral status. To claim that death is the loss of moral status amounts to conflating the cause with its consequence.”

Let me answer this objection with two counterarguments. Sometimes the cessation of an organism causes a loss of moral status, but whether it is the case might differ given the different senses of “organism” within various subdisciplines of biology. Consider the immunological account of individual organisms [25–27]. Suppose someone’s immune system is irreversibly destroyed by radiation. According to the immunological view, they are no longer considered an organism but remain conscious and capable of valuation for a while. Eventually, the complete destruction of their immunological system will lead to the loss of their capacity to value, as it relies on their cerebral hemispheres which will be destroyed without a functioning immune system.

So yes, death as the cessation of an organism in such a situation causes the loss of moral status, as the destruction of the immunological entity leads to the destruction of the valuing entity. However, despite not being considered an organism from the perspective of the immunological view on organismal individuality, the person with an irreversibly destroyed immune system remains conscious and capable of valuations. This makes them practically alive. It is not a mistake to claim that death (in a practically relevant sense) is the loss of moral status.

Second, although it is sometimes the case that death as the cessation of an organism is a cause of death as the loss of moral status, it also transpires that the termination of an organism occurs after the loss of moral status as in the typical case of brain death. Brain death is determined when the patient is on artificial life support, like a ventilator. From the perspective of immunological biology, such a patient is considered a living organism as the immunological system still functions, enabling the body to fight infections. However, despite the continued existence of the organism, the brain-dead patient will never regain the capacity to value anything, resulting in the irreversible loss of moral status. If the case of a brain-dead patient is not clear enough, a similar analysis can be applied to an artificially sustained decapitated body which shares similar characteristics in terms of consciousness and immunological capacities [112]. In summary, an entity's organismal status is only incidentally linked to its moral standing.

## Different views on moral status

The second objection to the view presented herein might be formulated in the following manner: “Your proposal to define death as an irreversible loss of moral status is susceptible to objection, similar to the criticism of death as the cessation of an organism’s functioning. The diversity of views on moral status implies that for a given physiological state, a patient may be considered both alive and dead in terms of moral standing.”

The objection underlines the main limitation of my proposal since there are in fact a diversity of views on moral status. Some propose identifying moral status with species membership, claiming only *homo sapiens* individuals possess moral status due to their belonging to the species [62, 113]. Others equate moral status with possessing the human soul [cf. 114]. Some identify moral status with something else, for example, sentience [77, 80, 115], being a person, rationality [68, 116], being an object that might be cared for [49, 117, 118], being an object that has a positive impact on ecosystem [119, 120], and being the object that someone believes that it has moral status [51]. Given that brain-dead body is genetically a member of the *homo sapiens* species but is no longer sentient, is it the case that it is morally both alive and dead?

I believe the objection can be answered successfully. Understanding death as a loss of moral standing, regardless of its grounding, is still preferable to viewing it as the cessation of an organism. Two reasons support this stance: the first is that moral status matters in everyday life, while organismal status does not (I allude here to the case of a conscious person who is not an organism but still has moral status – see the previous section). Moreover, moral status matters even though one does not have a genuine consensus about its criteria, even though the agreement here still needs to be worked out. It is discernible in the context of the abortion debate, which could be read in the following manner: in some countries or states, arguments showing that an early human fetus lacks moral status have proven to be convincing, while the opposite is true in some other countries or states.

The abortion controversy makes sense if read as a controversy on moral status, not if it were about the organismal status of a fetus. The fetus’ organismal status is irrelevant, much like the organismal status of myself or another. Whether I am an organism or a robot with consciousness (along with other non-relevant attributes such as skin color or sex status) should not affect how I am treated. Practically, what matters is if harm or good can happen to me, not my organismal status.

Another reason to favor conceiving death as the loss of moral status rather than as a cessation of an organism is the structural difference between pluralism in the philosophy of biology and the diversity of views on moral status in ethics. The pluralism in the philosophy of biology with its divergent verdicts on the organismal status of an individual, such as a termite for example, is a mainstream position in theoretical biology. The pluralism here is of that kind that biologists are aware of and even call for [16, 30]. They are aware that, for example, an individual organism is at the same time constituted by the termite’s body and protozoan living in its gut, given immunological theory [25, 26], and that protozoan is not a part of termite organism given the equally valid developmental concept of an organism [17, 22, 23]. Biologists specializing in immunology and development theory do not consider each other’s views to be false. There are different yet equally real ways to be an organism: one can be an organism as an immunological entity, as an evolutionary individual, as a developmental individual, and so on.

Unlike diverse biological perspectives on organisms, ethical views on moral status create genuine tension. Most ethicists in the ethical debate about moral status criteria believe that it is not merely a matter of perspective where all theories in normative ethics are equally valid. They do not endorse the idea that there are multiple equally real ways to have moral status and they would not consider, for example, the abortion of a fetus as both wrong and morally neutral.

Ethicists that defend different criteria of moral status hardly describe the difference between their positions as a matter of different equally valid ethical perspectives, so the disagreement between them persists on a general level. It is a disagreement about what ethics, all things considered, has to say. At the same time, biologists can accept all the concepts of an organism as valid within the divergent subdisciplines of biology.

In other words, the ethical disagreement is epistemological, not ontological and most ethicists believe that there is a single set of criteria for moral status that is valid universally, merely disagreeing regarding the choice. Biologists do not insist that only one of the multiple views on organisms developed within a particular branch of biology is supreme. Therefore, if one is looking for a universal and univocal criterion of death, it is more probable that one will find it on the grounds of the ethical analysis of moral status rather than in biology. In this paper, I have tried to move closer to this point by attempting to show the merits of the constructivist view on the nature of moral status.

## Nothing new! It is just sentience

The last objection to the constructivist view presented herein is that it is similar to other theories equating moral status with sentience, leading to the same old problems. Here are two clarificatory remarks in response.

First, let me emphasize that, according to constructivism, moral status does not rest entirely on sentience. Instead, it rests on the capacity to have affective attitudes, of which sentience is the most primitive example. As argued in the “Intuitionism and constructivism” section, humans share sentience with many animals. In contrast, few animals possess preferences, and practical reflection may be absent in all nonhuman animals. Since sentience is the most rudimentary affection, this part of our evaluative capacities is usually the last to be lost in the dying process.

However, consider someone with a congenital insensitivity to pain, a rare condition where one cannot feel physical pain, and for the sake of argument, assume one also cannot experience physical pleasure. Would my view imply that such a person has no moral status? Not at all. Even in this case, the person is capable of affection, being attracted to the fulfillment of their wishes and averse to desires not being fulfilled. The clearest example here might go like this: the person with congenital insensitivity to pain and pleasure wants to feel the joy of eating deep-dish pizza.

The constructivist approach does not rely solely on sentience as the criterion for moral status. It considers other forms of affection, which means insentient beings might still have moral status and be morally alive.

## Conclusion

In this article, I have proposed a constructivist concept of death based on the capacity for affective attitudes rather than organismal status. Such a concept overcomes the Transitivity Argument explaining why brain-dead patients are dead in a practically relevant sense and why end-stage dementia patients are alive. To sum up, end-stage dementia patients are alive because they persist as the same, albeit damaged, valuers as they used to be before dementia took its course. Honoring their advance directives to cease life-sustaining treatment is an instance of fulfilling a direct duty owed to these patients. When it comes to brain death, dishonoring the will of patients regarding the treatment of their brain-dead bodies is, according to the consequences stemming from my concept, either an instance of posthumous harm (which is done not to the brain-dead body but retrospectively to patients who used to be alive in the past as valuers) or a harm done to the society.

The merits of the constructivist theory of death include the following: it is not ad hoc and can be applied not only to resolve the brain death controversy but also in various contexts; it aligns with scientific understanding, and it avoids challenges akin to the organismal pluralism problem by striving to be rooted in the ethically privileged concept of moral status.

The most important limitation of the concept is that there is no consensus among ethicists that the capacity to have affective attitudes constitutes the sole basis for moral status. Yet this lack of consensus is structurally different than the lack of consensus among biologists on the unified concept of an organism, so it does not generate a similar problem to organismal pluralism. Moreover, this article might be read as a step forward on the path to reaching a consensus among ethicists on the right criterion of moral status.

## Footnotes

1 A reviewer of this article has suggested that a patient's biological life or death can be evaluated irrespective of her status as an organism in a way that does not generate a problem similar to the one associated with organismal pluralism. To reply to this objection, allow me to cite in full a paragraph from an article which I have co-authored with Adrian Stencel: “One could speak about death also in a different biological sense than the cessation of an organism, referring to the opposition between dead and living material. This is one of the most debated issues in biology and the philosophy of biology – one that is especially interesting when scholars try to find out whether viruses are alive or when they try to define life for astrobiological investigations. For both cases, figuring out whether something is living or dead is undoubtedly an important issue to drive research….However, in such a sense, it is beyond all controversy that brain-dead patients on artificial support are living since there are living organs, tissues, and cells in their bodies. They belong to the ‘life’ domain of the world. Therefore, in our opinion, the whole discussion about the status of brain-dead patients within the ‘biological-bioethical’ paradigm makes sense if it is a controversy about the existence of some cohesive biological individual such as an organism” [^30^, p. 149]. Yet, as the organismal pluralism problem shows, this interesting type of controversy is irresolvable.

2 Adrian Owen and other researchers have shown that around 20% of patients diagnosed as being in a vegetative state actually have locked-in syndrome [95]. Locked-in syndrome is a condition where one is aware of surroundings but unable to respond intentionally to stimuli. Owen’s neuroimaging techniques revealed preserved fronto-parietal activity in some clinically diagnosed vegetative state patients, indicating consciousness. Hence, diagnosing death in a persistent vegetative state (like Veronica’s) is uncertain.
